# Collaborative model of care between Orthopaedics and allied healthcare professionals in knee osteoarthritis (CONNACT): study protocol for an effectiveness-implementation hybrid randomized control trial

**DOI:** 10.1186/s12891-020-03695-3

**Published:** 2020-10-16

**Authors:** Bryan Yijia Tan, Michelle Jessica Pereira, Su-Yin Yang, David J. Hunter, Soren Thorgaard Skou, Julian Thumboo, Josip Car

**Affiliations:** 1 Department of Orthopaedic Surgery, Woodlands Health Campus, National Healthcare Group, Singapore, Singapore; 2 Singapore General Hospital, Singapore City, Singapore; 3 Health Services Outcome Research, National Healthcare Group, Singapore City, Singapore; 4 Tan Tock Seng Hospital, Singapore City, Singapore; 5 Institute of Bone and Joint Research, Kolling Institute, University of Sydney and Rheumatology Department, Royal North Shore Hospital, Sydney, Australia; 6 Department of Sports Science and Clinical Biomechanics, Research Unit for Musculoskeletal Function and Physiotherapy, University of Southern Denmark, Odense M, Denmark; 7 Department of Physiotherapy and Occupational Therapy, Næstved-Slagelse-Ringsted Hospitals, Slagelse, Denmark; 8 Lee Kong Chian School of Medicine, Nanyang Technological University, Singapore City, Singapore

**Keywords:** Study protocol, Effectiveness-implementation hybrid, Mixed methods, Knee osteoarthritis, Model of care, Randomized control trial

## Abstract

**Background:**

Knee Osteoarthritis (OA) is a leading cause of global disability. The **Collaborative Model of Care between Orthopaedics and Allied Healthcare Professionals (CONNACT)** Model of Care (MoC) was developed by optimizing evidence-based non-surgical treatments to deliver value-based care for people with knee OA. The primary aim of this study is to determine the clinical effectiveness of the CONNACT MoC (3 months) compared to usual care. The secondary aims are: a) To determine the cost-effectiveness and b) To develop an evaluation and implementation framework to inform large scale implementation for this MoC.

**Methodology:**

Type 1 Effectiveness-Implementation Hybrid Trial using an explanatory sequential mixed-method approach. The study consists of 3 components. The first component is the pragmatic, parallel-arm, single-blinded randomized control trial. Inclusion criteria are patients with knee OA based on the National Institute of Health and Care Excellence (NICE) criteria with radiographic severity of greater than Kellgren-Lawrence 1, and Knee Injury and OA Outcome Score (KOOS_4_) of equal or less than 75. Exclusion criteria include other forms of arthritis, history of previous knee arthroplasty or wheelchair-bound patient. KOOS_4_ is the primary outcome measure at 3 months, 6 months and 1 year. Secondary outcomes include KOOS individual subscales, quality of life scoring, functional performance, global, diet and psychological related outcomes. The second component is an economic evaluation of the cost-effectiveness of the CONNACT MoC using a societal perspective. The third component is an implementation and evaluation framework using process evaluation under the RE-AIM framework using a mixed-method approach. Sample size of 100 patients has been calculated.

**Discussion:**

CONNACT MoC is a complex intervention. In line with the MRC guidance for developing and evaluating complex interventions, a pilot feasibility study was completed and a comprehensive approach including an RCT, economic evaluation and process evaluation is described in this study protocol. Results from this study will help clinicians, healthcare administrators and policymakers guide the sustainable and effective implementation of the CONNACT MoC for knee OA and serve as a basis for similar multidisciplinary MoC for chronic degenerative musculoskeletal conditions to be developed.

**Trial registration:**

Clinicaltrials.gov Identifier: NCT03809975. Registered January 182,019.

## Background

The world is experiencing a rapidly aging population, and with it an age-related increase in chronic musculoskeletal (MSK) disorders. Based on the 2010 Global Burden of Disease Study, MSK disorders account for the largest cause of disability around the world and in particular, osteoarthritis (OA) was ranked the 11th highest global cause [1]. International guidelines are consistent in their treatment recommendations for knee OA with individualized lifestyle changes, especially exercise and weight loss programs, highlighted as first-line management [2, 3]. A stepwise approach is recommended where surgery is considered when non-surgical treatment fails. Despite all this, studies report that more than half of the patients from established healthcare systems around the world such as Australia and Canada are not receiving optimal non-surgical treatment [4–6].

Surgery is often a result not from a failure of non-surgical treatment but the failure of the healthcare system to provide adequate and effective non-surgical treatment. With knee arthroplasty rates expected to rise rapidly [7], the literature suggests that at least a quarter of knee arthroplasties could have been avoided through optimal non-surgical treatments [8]. While knee arthroplasty surgery is an effective option for knee OA, it should be applied only when all non-surgical treatment options have been exhausted as it is costly and not without risks and complications [9].

There is an urgent need for new models of care (MoC) for knee OA developed by optimizing evidence-based non-surgical treatments to deliver value-based care. There has been a paradigm shift globally, moving from an acute episodic type treatment that was generally associated with OA to a chronic disease MoC [10]. Over the past decade, several models of care from around the world have been developed including the Good Life with OA in Denmark (GLA:D) program and the OA Chronic Care Program (OACCP) program from Australia [11]. Building from the well-established principles of these programs, the **Collaborative Model of Care between Orthopaedics and Allied Healthcare Professionals (CONNACT)** Model of Care (MoC) of knee OA was developed. CONNACT is a community-based, multidisciplinary 12-week program that uniquely uses an individualized approach based on a triaging criterion to tailor the treatment to each patient in line with the “*right care*, delivered at the *right time*, by the *right team*, in the *right place*, with the *right resources*” philosophy coupled with a strong emphasis on patient activation and self-management strategies to promote long term sustainable behavioural change.

The Medical Research Council (MRC) Guidance on developing and evaluation such complex interventions was utilized in the CONNACT development, evaluation and eventual planned implementation [12]. A feasibility study using a pilot randomized trial design was conducted to determine the feasibility of a full randomized controlled trial (RCT) evaluating the CONNACT MoC as the first step [13]. Results from the feasibility study were instrumental in the design of this study protocol.

### Aim

The primary aim of the study is to evaluate the clinical effectiveness (pain, function, quality of life) of the CONNACT MoC as compared with usual care in patients with knee OA.

The secondary aims are to:

1. Evaluate the cost-effectiveness of the CONNACT MoC for patients with knee OA
2. Develop an implementation and evaluation framework to inform large scale implementation of CONNACT MoC

We hypothesize that patients with knee OA who undergo the CONNACT program will have better pain, function and quality of life scores 12 months after initiating the program compared to patients who undergo usual care.

## Methodology

### Design

The study is an Effectiveness-Implementation Hybrid Trial which combines both an effectiveness and implementation component [14, 15]. It has been increasingly recognized that implementation is the main challenge in many models of care. An implementation gap, also known as the “valley of death” exists between what current literature recommendation and translation to actual clinical practice [16]. Knee OA is a clear situation where this “valley of death” has happened. As a result, there has been a greater emphasis on incorporation of an implementation element in studies to enhance dissemination with hybrid trials increasingly being conducted [17].

This will be a type 1 hybrid trial where the emphasis and primary aim is to evaluate effectiveness through a pragmatic randomized trial under real-world conditions and its secondary aim is to understand the context of implementation through a mixed-method, process orientated approach. The pragmatic nature of the study was guided by the Pragmatic Explanatory Continuum Indicator Summary (PRECIS-2) tool [18].

The study will be conducted as a single centre pragmatic, parallel-arm, single-blinded RCT using a mixed-method approach comparing the CONNACT multidisciplinary personalized community-based MoC and the current MoC. The Standard Protocol Items: Recommendations for Interventional Trials (SPIRIT) [19] model (Additional file 5) and the OA Research Society International (OARSI) clinical trial recommendation on the design and conduct of clinical trials for knee OA [20] guided the development and reporting of the trial protocol. The findings of the trial will be reported according to the Consolidated Standards of Reporting Trials (CONSORT) 2010 [21] guidelines for reporting parallel group randomized trials. The study will utilize an explanatory sequential mixed methods design where the qualitative data through the use of surveys and interviews will be used to interpret and provide context for the quantitative results.

### Ethics approval

Ethics approval has been obtained from the Institution Review Board (IRB) prior to the conduct of the study (NHG DSRB Ref: 2018/00408). Any protocol modification will be communicated to IRB in a timely manner. Random audits will be performed by the IRB.

### Participants

We will recruit patients based on the inclusion and exclusion criteria outlined in Table 1.

**Table 1:** Inclusion and Exclusion Criteria

Inclusion Criteria (all 4 must be present)	Exclusion Criteria
National Institute of Health and Care Excellence (NICE) clinical criteria for knee OA [22] Age ≥ 45 years old **and** Has activity related knee pain **and** Has either no morning knee-related stiffness or morning stiffness than last no longer than 30 min	Alternative diagnosis to knee OA e.g. referred pain from the spine or hip
Radiographic severity of knee OA, Kellgren-Lawrence Score [23] > 1	Other forms of arthritis e.g. inflammatory, post-traumatic
Knee Injury and OA Outcome Score [24] (KOOS_4_)* ≤ 75	Inability to comply with study protocol e.g. cognitive impairment
Community ambulator with or without walking aid	Previous knee arthroplasty
	Wheelchair-bound patients
	Medical conditions that will medically interfere with study involvement e.g. decompensated heart failure, stroke, end-stage renal failure

* KOOS_4_ is a composite score of the mean of four of the five subscale scores from the Knee Injury and OA Outcome Score (symptoms, pain, function from daily living and quality of life)

### Components of the CONNACT study

1. Randomized Controlled Trial
2. Economic evaluation
3. Implementation and Evaluation Framework

## COMPONENT 1: randomized controlled trial

### Trial procedure

**Fig. 1:**
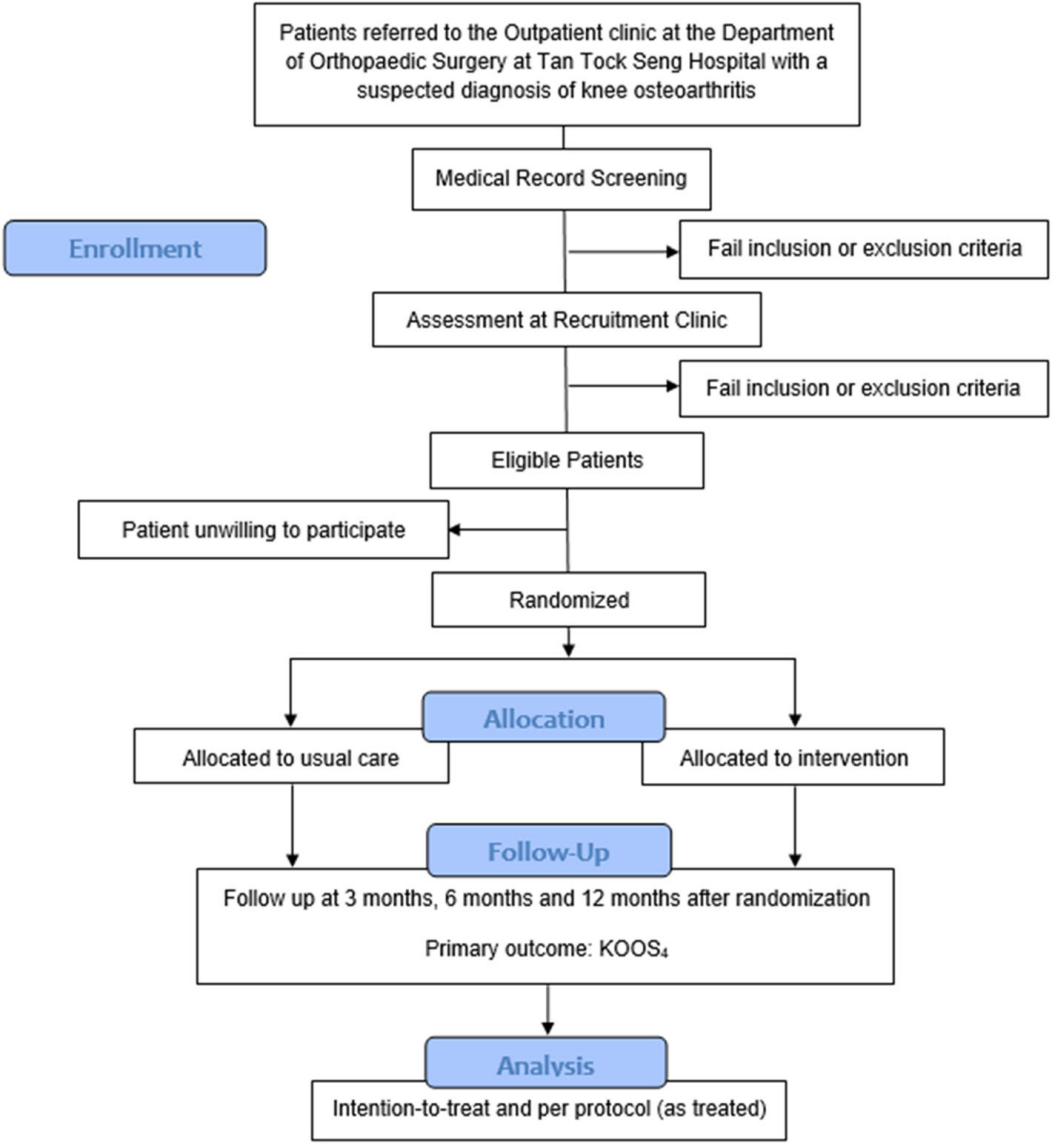
Randomized Control Trial Structure

The overall flow for the RCT is outlined in Fig. 1 based on the CONSORT guidelines. Patients who are referred by a primary healthcare or emergency medicine doctor to the Outpatient clinic at the Department of Orthopaedic Surgery at Tan Tock Seng Hospital, a tertiary referral centre in Singapore with a suspected diagnosis of knee OA will be screened based on the inclusion and exclusion criteria presented in Table 1. All referral letters are screened based on electronic medical records.

Patients who are eligible based on initial screening are invited to attend a recruitment clinic where they will be assessed by the study team and invited to participate in the study if they meet all the inclusion and exclusion criteria. Informed written consent will be obtained. Patients will be randomized into the intervention or usual care arm after collection of baseline data. Recruitment clinics will be carried out at the Tan Tock Seng Hospital Specialist Outpatient Clinic.

### Randomization and concealment of allocation

Patients who consent to participate will be randomized (1:1 allocation ratio) between the intervention and usual care using a stratified permuted block randomization method using block sizes of 4,6 and 8. Stratification is based on gender to ensure equal distribution in both groups. The allocation sequence is generated by an independent statistician a priori and will be kept concealed from the study team. Randomization will be done using the REDCap randomization module based on the allocation sequence and allocation will be locked once assigned. Randomization and intervention allocation will only be performed by the study team after the patient is counselled fully about the study and provides informed consent.

### Intervention – CONNACT model of care

The development and core principles of the CONNACT MoC has been described as part of the pilot study [25]. Grounded from a throughout literature search on the best practices in knee OA care [3, 26], a review of successful programs [11], international collaborations and the process evaluation patient interviews from the pilot study has allowed the CONNACT MoC to be further refined and contextualized.

The CONNACT MoC consists of a 12-week community based, individualized, multidisciplinary intervention for knee OA. In summary, the core principles are firstly a chronic disease multidisciplinary model emphasizing self-management and long term sustainable behavioural change, secondly, care stratification using a triage criterion and thirdly, implemented through a grounded community-based setting.

Table 2 outlines the intervention summary, including each component, triaging criterion, intervention principles and delivery. A full description of the intervention in accordance with TIDieR [27] and CERT [28] reporting guidelines is available in the Additional file 1. Further rationale and details for the triaging criterion and the self-management philosophy underpinning the CONNACT MoC are elaborated below.

**Table 2:** Intervention Summary

Intervention Component	Criteria to receive intervention	Healthcare Professional	Treatment Principles	Delivery Format
Exercise Therapy	All patients	Physiotherapist	American College of Sports Medicine (ACSM) [29] and Neuromuscular Exercise (NEMEX) [30] guidelines	Group sessions × 8
Clinical Assessment and Education	All patients	Orthopaedic Surgeon Psychologist Social Worker	Clinical and Radiological Assessment Pharmacological Intervention “Expert” Patients	Group Education sessions × 2 Support Group session x 1
Dietetics and Nutrition	BMI > 23.5	Dietician	Dietary intervention to increase dietary-related nutrition knowledge and self-efficacy for effective weight loss [31]	Group sessions × 3
Psychological support	PHQ-4 > 5 or PEG > 4 on all scales or PAM < 3	Psychologist Social Worker	Acceptance and Commitment Therapy (ACT) principles [32, 33] Patient Activation [34] Pain Management Coping Strategies and improving compliance to behavioural modifications	Group sessions × 3

#### Triaging criteria development

The 3 measures that were chosen for psychological intervention triaging criteria were Patient Health Questionnaire-4 (PHQ-4) [35], and **P**ain Intensity, **E**njoyment of life and **G**eneral Activity (PEG) [36] and Patient Activated Measure (PAM) [37].

PHQ-4 and PEG were chosen in light of the significant impact of psychological conditions (anxiety, depression) and pain intensity and interference in predicting outcomes in OA patients [38, 39]. The **P**ain Intensity, **E**njoyment of life and **G**eneral Activity (PEG) [36] is a brief, 3-item assessment of pain intensity (1item) and pain interference (2 items) derived from the Brief Pain Inventory (BPI) [40]. The PEG measures pain intensity on a numerical rating scale of ‘0’ (no pain) to ‘10’ (pain as bad as you can imagine) and pain interference with general activity and enjoyment of life also on a ‘0’ (does not interfere) to ‘10’ (completely interferes) scale. Provisional benchmarks have been set to identify clinically meaningful improvements that can help differentiate between treatment “responders” and “non-responders” at the start of a clinical trial [41] Suggestions that symptom reduction of 30% or more indicating moderately important improvements and symptom reduction of 50% or more considered “substantial improvements”. Taking reference from these guidelines, a cut-off score of “5” indicating moderate symptoms for pain, physical functioning was used to decide for psychological intervention.

The Patient Health Questionnaire-4 (PHQ-4) [35] is a brief 4-item screening assessment of depression and anxiety symptoms. With anxiety known to exert an independent effect on functioning separate from depression, screening for both depression and anxiety has been thought to be necessary [42]. The first 2 items from the PHQ-4 are taken from the original 9 item Patient Health Questionnaire (PHQ-9) and the next 2 items taken from the first 2-items on the General Anxiety Disorder (GAD-7) questionnaire. The PHQ-4, therefore, forming a composite score of depression and anxiety. Responses on all 4 items are scored as “0” (not at all), “1” (several day”, “2″ (more than half the days) and “3″ (nearly every day). A score of more than 5 indicates at least a moderate psychological distress from depression and/or anxiety and thus was used as cut off for psychological intervention.

Patient activation is defined as an individual’s propensity to engage in adaptive health behaviour that may lead to improved outcomes. Activation levels are measured by the Patient Activated Measure (PAM) [37], a validated questionnaire that looks at knowledge, skills and confidence in managing health. Through the PAM score, patients can subsequently put into 4 groups, level 1 (poorly activated) to level 4 (highly activated). There has been increasing evidence in the literature that PAM scores are modifiable through targeted intervention and that high PAM scores have been associated with more satisfaction with healthcare services, better self-management behaviour and improved health outcome [43, 44]. As such, PAM levels of 1 and 2 were chosen as a triaging criterion for targeted psychological intervention in an effort to boost activation levels.

Body Mass Index will be used as a triaging criterion for dietetic intervention. A lower cut off of BMI 23.5 has been recommended for Asians [45] and will be used as a cut-off.

#### Self-management philosophy

Self-management is a key foundation for the CONNACT MoC. This will be achieved through several methods. Firstly, through the program content. The education and psychology classes grounded in the principles and psychological model of Acceptance and Commitment Therapy (ACT) [32, 33] and Patient Activation [34] strategies which have been shown to the effective. In particular, there has already been evidence to show the effectiveness of ACT in the local chronic pain population in Singapore [46].

Secondly, through the program delivery format. The program’s duration of 12 weeks is based on current evidence of the time taken for a habit to form [47]. The program is structured as a weekly class with a gap of 3 weeks between week 8 and week 12 factored in to allow patients the opportunity to incorporate these behavioural modifications as part of their daily routine before returning for the final session on week 12. In addition, after each session, patients will be given “homework” and exercises to perform at home and are reviewed at the next class. This combination of class-based and home-based individual treatment has been shown to be effective in knee OA [48]. In addition, group or class-based interventions have also been shown to be more effective compared to pure individual interventions in promoting physical activity through cohesion and peer support [49]. A flexible post-intervention program will be available for patients who would like to continue to exercise together in a group and social network platforms e.g. Whatsapp group chats or Facebook groups will also be utilized to facilitate patient group interaction throughout and post-intervention. In addition, a support group session 3 months after program completion will be arranged for reinforcement for key concepts and continued promotion of group interaction.

Thirdly, through the use of “expert patients”. These “expert patients” are patients who have previously completed and benefitted from the CONNACT program. They will be invited to volunteer and share their experiences and advice as part of the education class to serve as a role model for patients. “Expert patients” been successfully incorporated in other knee OA programs before [30].

### Usual care

Usual care constitutes a referral to the outpatient physiotherapist at the tertiary hospital where patients are usually seen 1–2 weeks post referral. The physiotherapist would conduct an assessment and recommend a variety of lifestyle modifications and exercise therapy. The type of exercises and number of physiotherapy sessions were at the discretion of the patient and the physiotherapist.

### Outcome assessment and blinding

Outcome measures will be measured by blinded outcome assessors. The outcome assessors will be either physiotherapy interns or research assistants. All outcome assessors will receive training prior to study initiation to ensure good inter- and intra-observer reliability, particularly for the functional performance testing. Patients will be instructed not to reveal their allocation to the outcome assessors. Outcome assessment will be conducted either at the community rehabilitation facility or tertiary hospital setting. Choice of outcome assessment location will be carefully monitored to ensure that outcomes assessors were not able to deduce the treatment arm.

### Outcomes

The choice of outcome measures is based on the OARSI guidelines for lifestyle diet and exercise clinical trials in OA [50]. The recommended core outcomes are pain, physical function, global patient assessment and mobility. Additional outcomes include health-related quality of life and global physician assessment. The outcome measures will be collected at baseline, 3, 6 and 12 months. Table 3 summarizes all the outcome measures and Additional file 4 outlines the different data collection steps based on the SPIRIT guidelines.

**Table 3:** Outcome Measures Overview

Effectiveness Measures				Compliance and Adherence Measures
Physical Function	Dietetics	Psychology	General	
KOOS_4_	Body Mass Index (BMI)	Patient Health Questionnaire 4 (PHQ-4) [35]	Quality of Life EQ-5D [51]	Appointment default rate
KOOS individual subscales	Semi-Quantitative Food Frequency Questionnaire (FFQ) [52]	Patient Activation Measure (PAM) [37]	Global Perceived Effect (GPE) [53]	Exercise Adherence Questionnaire
Functional Performance 1. Timed up-and-go 2. 4 × 10 m face paced walk test 3. 4-stair climb test 4. 30s chair stand		Pain, Enjoyment, General Activity Scale (PEG) [36]	Patient Acceptable Symptom Score (PASS) [54]	Sports Injury Rehabilitation Adherence Scale (SIRAS) [55]
UCLA activity score [56]			Analgesia Consumption [57]	
			Adverse events	

### Baseline measures

The following baseline measures will be collected.

1. Demographic – age, gender, race
2. Socioeconomic status – education level, housing status, employment details
3. Co-morbidities and functional status – Charlson comorbidity index [58], Barthel Index for Activities of Daily Living [59], Parker Mobility Score [60]
4. Knee symptoms and duration
5. Radiographic severity of knee OA based on the Kellgren Lawrence Scale [23]

### Primary outcome

The primary outcome measure will be the mean of four of the five subscale scores from the Knee Injury and OA Outcome Score (KOOS_4_). KOOS contains 5 domains of questions, namely symptoms, pain, function (daily living), function (sports, recreational activities) and quality of life [24]. Consistent with other studies with a similar population of elderly patients with knee OA, the function (sports, recreational activities) subscale were deemed to be less relevant for this population and the remaining 4 domains were combined to form a composite score [61]. The KOOS score has previously been validated in Singapore [62].

### Secondary outcomes

The secondary outcomes have been classified into effectiveness and compliance outcomes. The effectiveness measures have been further subdivided based which aspect of the treatment it is likely to have the greatest impact on.

#### Physical function outcomes

Secondary physical function outcome outcomes included KOOS individual subscales, functional performance and ULCA activity score. The choice of functional performance tests was based on the recommended OARSI performance test for functional testing in OA [63]. A 4 × 10 m fast-paced walk test, timed up-and-go, 4-stair climb test and 30-s chair stand were chosen to encompass the key domains of functional activities from sit-to-stand, walking short distances, stair negotiation and ambulatory transitions. The ULCA activity score is a validated score that is recommended for use in patients with hip or knee OA [64].

#### Dietetics outcomes

In addition to Body Mass Index, Food Frequency Questionnaire (FFQ) will be collected to monitor for change in dietary habits. Modification to the original Food Frequency Questionnaire (FFQ) was performed to reduce the length and thereby responder burden and adapt it based on local dietary practices. Scoring was developed by weighting fat/sugar/fibre content of the particular food item based on the energy and nutrient composition reported by the Singapore Health Promotion Board (http://focus.hpb.gov.sg/eservices/ENCF/). Modified FFQ will only be done for patients who have a BMI > 23.5.

#### Psychological outcomes

The Pain Interference Scale (Pain intensity (P), interference with enjoyment of life (E), interference with general activity (G))(PEG) [36] is a 3 item scale developed and validated from the Brief Pain Inventory(BPI) for chronic pain measurement, particularly in chronic musculoskeletal patients. The Patient Health Questionnaire-4 (PHQ-4) [35] is a validated questionnaire that allows measurement of both anxiety and depression, both common psychological conditions associated with poorer quality of life in OA patients [38]. Patient activation is defined as an individual’s propensity to engage in adaptive health behaviour that may lead to improved outcomes [43, 44]. Activation levels are measured by the Patient Activated Measure (PAM) [37], a validated questionnaire that looks at knowledge, skills and confidence in managing health.

#### General outcomes

Quality of Life will be measured using the EQ-5D, both the descriptive index and EQ-VAS [51]. The EQ-5D value set that has been validated for the Singapore population using a time trade-off method and was used to calculate utility values [65]. It will also be used in the computation for Quality of Life Years (QALYs) and eventual cost-effectiveness.

Analgesia consumption will be measured using the Cumulative Analgesia Consumption Scale (CACS) which measures both the quantity and potency of the analgesia based on WHO analgesia ladder over a 1 week period [57].

Global perceived effect (GPE) [53] will be assessed with the question: With respect to your knee, how would you describe yourself now compared to before the treatment?? Answered on a seven-point Likert scale ranging from ‘Improved, an important improvement’ to ‘Worse, an important worsening’. Satisfaction with current knee function will be assessed by the Patient Acceptable Symptom State (PASS) [54] using the question: “When you think of your knee function, will you consider your current condition as satisfying? Answered by ‘yes’ or ‘no’. Participants not satisfied with current knee function will be asked to complete a second single-item question, relating to treatment failure (TF) at the 12-month follow-up: ‘Would you consider your current state as being so unsatisfactory that you think the treatment has failed?’. Answered by ‘yes’ or ‘no’.

Any adverse events (AE) and serious adverse events (SAE) will be collected during all follow-up visits using open-probe questioning to ensure all AE are recorded. SAE will be defined as any AE that results in hospitalization or is life-threatening. Participation in the trial for the subject will cease if a SAE occurs. Undergoing an indicated surgical procedure such as a knee arthroplasty for knee OA due to failure of non-surgical treatment will not be considered an AE. In the event of more than 3 SAE, decision to stop the trial will be considered however the final decision will be made by the Principal Investigator (PI) in conjunction with the ethics and clinical board. Provision for post-trial care and compensation will be made for subjects who suffer harm during the course of the trial.

#### Compliance and adherence

Compliance and adherence are key aspects in any exercises-based intervention for MSK conditions and having compliance measures allows for a more accurate interpretation of the results and form a significant part of the process evaluation [66]. Unfortunately, there is no specific measure for exercise adherence that has been proven to be high quality, relevant and acceptable [67]. In order to give a holistic picture to adherence, the Sports Injury Rehabilitation Adherence Scale (SIRAS) [55] will provide the healthcare providers perspective while a self-administered questionnaire for patients has been designed to obtain patient’s input (Additional file 2). SIRAS is a 3-item instrument that assesses patient’s intensity to exercise, the extent to which instructions were followed and receptiveness to change during each rehabilitation session. SIRAS will be done after each physiotherapy session and the exercise adherence questionnaire will be done at 3, 6 and 12 months. These adherence measures will only be performed in the intervention arm patients due to the variability of treatment delivery in the usual care arm. Non-compliance will be defined as less than 75% attendance of the prescribed treatment in the intervention arm. For usual care, the number of physiotherapy sessions will be at the discretion of the patient; thus, no non-compliance cut off limit will be set.

### Sample size calculation

The sample size needed to detect a 10-point difference between the intervention arm and usual care arm in KOOS_4_ was 41 patients in each arm based on a power of 90%, *p*-value of 0.05 (two-sided) and a standard deviation of 14. The anticipated population KOOS_4_ and standard deviations were based on the initial pilot study [25] done and the minimally clinically important difference on 10-points (MCID) for KOOS [24]. In order to account for a missing data rate of 20%, 100 patients will be recruited for the study.

### Analysis plan

The primary endpoint in this study is KOOS_4_ at 12-month follow-up. Results will be analysed by intention-to-treat (ITT) principle. However, data will also be analysed by per-protocol (PP) approach to account for protocol violations such as patients who were deemed not compliant to treatment or patients who underwent a surgical procedure of the knee due to treatment failure during the course of the study.

Descriptive frequency analysis will be used for baseline characteristics. For continuous variables, the mean and standard deviation will be reported and for categorical variables, the frequencies and percentages will be reported. Between-group comparisons of change from baseline to 1-year follow-up in the primary and secondary continuous outcomes will be analysed using a generalized linear mixed model (GLMM). Testing for normality of distributions of outcomes will be based both on the Shapiro-Wilks test and a visual analysis of the histogram plot. Categorical secondary outcomes will be analysed using the ordinal logistic regression function under GLMM. A two-sided *p*-value less than 0.05 will be considered as statistical significance. An analysis will be done on the nature of the missing data to determine if the data was missing at random or a systemic bias was present resulting the missing data.

### Data management

All data will be monitored by the PI or the study team, independent on the study sponsor. Data quality measures include queries to identify outliers and missing data. A unique identifier will be assigned to each subject after enrolment to ensure patient confidentiality. Data will be collected and stored on the Research Electronic Data Capture (REDCAP) system which is a widely used and secure web application for clinical data management in research. The REDCAP system is password protected and will only be accessible by the study team.

## COMPONENT 2: economic evaluation

### Aim and study design

The aim of the economic evaluation is to evaluate the cost-effectiveness of a personalized, community based 12-week multidisciplinary program for patients with knee OA. The Panel on Cost-Effectiveness in Health and Medicine recommends the use of a societal perspective to ensure that potentially important indirect costs such as productivity and caregiver cost would not be omitted [68]. The study will be conducted from a societal perspective to determine the cost-effectiveness of the intervention [68]. Results from the economic evaluation will be reported based on the Consolidated Health Economic Evaluation Reporting Standards (CHEERS) Statement [69].

### Cost estimation and outcome measurement

Cost data will be collected via hospital administrative databases and patient-reported questionnaires to estimate direct medical, direct non-medical and indirect costs. Indirect costs include health-related productivity loss due to knee OA [70] from absenteeism and presenteeism, measured with the Work Productivity and Activity Impairment Questionnaire (WPAI) [71]. The scope of the cost data collection was based on the validated OA Cost and Consequences Questionnaire (OCC-Q) [72] and adapted to the Singapore context to ensure that all relevant sources of cost were collected. The cost questionnaire used can be found in Additional file 3. Cost data will be collected at 3 monthly intervals till 1-year follow-up.

The primary measure of health benefit will be Quality of Life Years (QALYs) measured using the EQ-5D [51]. The incremental cost-effectiveness ratio (ICER) over the trial period of this multidisciplinary non-surgical community-based program for knee OA compared to usual care will be determined.

## COMPONENT 3: implementation and evaluation framework

### RE-AIM framework

The RE-AIM framework has been proposed as an appropriate model to strengthen the case for policy change and implementation for knee OA [11]. A recent study in Canada in 2018 used the RE-AIM model to evaluate the cross-cultural adaption and implementation of the GLA:D program [30] for hip and knee OA [73]. Table 4 outlines the definition and proposed data collection based on the RE-AIM criteria.

**Table 4:** RE-AIM framework and Data Collection

Dimension	Definition	Data Collection
Reach	The absolute number, proportion, and representativeness of individuals who are willing to participate in a given initiative, intervention, or program.	CONSORT flow Eligibility Log and reasons for non-participation Reasons for withdrawals Qualitative methods
Effectiveness	The impact of an intervention on important outcomes, including potential negative effects, quality of life, and economic outcomes.	Primary and secondary outcomes Adverse outcomes Economic Evaluation
Adoption	The absolute number, proportion, and representativeness of settings and intervention agents (people who deliver the program) who are willing to initiate a program.	Qualitative Methods with Healthcare Professionals
Implementation	At the setting level, implementation refers to the intervention agents’ fidelity to the various elements of an intervention’s protocol, including consistency of delivery as intended and the time and cost of the intervention. At the individual level, implementation refers to clients’ use of the intervention strategies.	Compliance (attendance logs, SIRAS, exercise adherence questionnaire) Process evaluation through Qualitative methods
Maintenance	The extent to which a program or policy becomes institutionalized or part of the routine organizational practices and policies. Within the RE-AIM framework, maintenance also applies at the individual level. At the individual level, maintenance has been defined as the long-term effects of a program on outcomes after 6 or more months after the most recent intervention contact.	12-month outcome measures Process evaluation through Qualitative methods

A process evaluation will be embedded in the study. The process evaluation is key in understanding the why and the how of any intervention by examining its implementation, mechanisms of impact and contextual factors. The MRC has developed a set of guidelines for the conduct of process evaluations [74]. MRC recommends a basic framework for process evaluation with a different emphasis at each stage of the study. In the pilot phase, the key would be understanding the feasibility and intervention design optimization. In the main trial phase, the focus is on the fidelity of actual delivery, context and mechanism of impact. Context includes anything external to the intervention that may act as a barrier or facilitator to its implementation. The mechanism of impact seeks to identify the potential causal pathways that resulted in the changes seen.

In addition, the Global Alliance for Musculoskeletal Health (GMUSC) have proposed a framework to help individuals and organization with the planning, implementation and evaluation of models of care (MoC) in MSK health [75]. The implementation and evaluation framework in this study will utilize all 3 frameworks by using RE-AIM framework to guide the focus areas, the MRC process evaluation to understand the underlying mechanisms and the GMUSC framework to ensure system-level relevance for scalability. Results from the study will be reported according to the Standards for Reporting Implementation Studies (StaRI) [76].

### Qualitative study methodology

A combination of deductive and iterative approaches will be used. A literature review will be conducted to generate themes for the study topic guide. A combination of questionnaires and semi-structured interviews will be conducted based on the topic guide. Purposeful sampling with both healthcare professionals and patients involved in the study will be used to identify appropriate participants for interviews.

Interviews will be conducted by an independent assessor with previous experience in qualitative research not directly involved in the care of the patients. Any interviewer pre-existing bias will be identified and recorded. Transcripts and audio recordings will be stored digitally and analysed using a qualitative analysis program to code data, link concepts, examine similarities and differences and review patterns and themes. Coding will be done iteratively.

Themes emanating from transcripts will be identified through a framework method approach done by 2 independent researchers supervised by a senior researcher with significant experience in qualitative research to ensure inter-observer reliability. Using the framework method, the data is reduced through a matrix comparing categories of data [77]. The framework method has been shown to be an appropriate method for the evaluation of multidisciplinary complex intervention. Data will be sampled till saturation. Results will be reported according to the Consolidated criteria for reporting qualitative research (COREQ) guideline [78].

## Discussion and conclusion

A rapidly aging global population has resulted in an OA epidemic that is currently being poorly managed on a global scale. CONNACT is a community-based, multidisciplinary 12-week program that uniquely uses an individualized approach based on a triaging criterion to tailor the treatment to each patient coupled with a strong emphasis on patient activation and self-management. Traditional MoC for knee OA utilize a step-wise approach where all patients get baseline education, exercise and weight loss advice before gradually moving up the treatment ladder if treatment is unsuccessful [79]. In line with the “*right care*, delivered at the *right time*, by the *right team*, in the *right place*, with the *right resources*” philosophy, CONNACT proposes the use of an individualized approach through the use of a triaging criterion.

A complex intervention is defined by the MRC as an intervention that contains several interacting components [12]. There are several dimensions of complexity that can exist including the interaction between components of the control and intervention arms, difficulty of behaviours change required to deliver or receive the intervention, number of groups targeted, variability of outcomes to the degree of flexibility of intervention permitted. With rapidly evolving healthcare systems, complex interventions are being developed in the common drive to provide cost effective, sustainable care for patients. However due its inherent nature, there are issues in describing, developing, documenting and implementing complex intervention [80].

CONNACT MoC is a complex intervention that aims to promote long term sustainable behavioural change for patients with knee OA. In line with the MRC guidance for developing and evaluating complex interventions, building from a pilot feasibility study, this study protocol describes a comprehensive approach including an RCT, economic evaluation and process evaluation using a mixed method approach. The primary aim of this study is to determine the clinical effectiveness of the CONNACT MoC compared to usual care. The secondary aims are: a) To determine the cost-effectiveness and b) To develop an evaluation and implementation framework to inform large scale implementation for this MoC. The study will utilize an explanatory sequential mixed methods design where the qualitative data will be critical in providing context for the quantitative results allowing for a clearer interpretation.

Results from this study will help clinicians and healthcare administrators work side by side to avoid the “valley of death”, bridge the gap between research and clinical practice, guiding the sustainable and effective large-scale implementation of the CONNACT MoC for knee OA. In addition, CONNACT can serve as a basis for similar multidisciplinary complex MoC for chronic degenerative musculoskeletal conditions to be developed in line with the MRC guidelines.

## Supplementary information

Additional file 1. Appendix 1 – Intervention Description. (DOCX 26 kb)

Additional file 2. Appendi× 2 – Patient Reported Exercise Compliance Questionnaire. (PDF 265 kb)

Additional file 3. Appendi× 3 – Cost Questionnaire. (DOCX 43 kb)

Additional file 4. Appendix 4 – SPIRIT diagram. (DOC 74 kb)

Additional file 5. Appendix 5 – SPIRIT checklist. (DOC 124 kb)

## Data Availability

Not applicable.

## Abbreviations

CONNACT: Collaborative Model of Care between Orthopaedics and Allied Health Professionals Trial
MSK: Musculoskeletal
OA: Osteoarthritis
MoC: Model of Care
RCT: Randomized Controlled Trial
NICE: National Institute of Health and Care Excellence
ACSM: American College of Sports Medicine
NEMEX: Neuromuscular Exercise
OARSI: OA Research Society International
KOOS: Knee Injury and OA Outcome Score
EQ-5D-5L: EuroQol 5 dimensions 5 level
BMI: Body Mass Index
PHQ-4: Patient Health Questionnaire 4
PEG: Pain, Enjoyment, General Activity Scale
ACT: Acceptance and Commitment Therapy
FFQ: Food Frequency Questionnaire
ITT: Intention-to-treat
PAM: Patient Activation Measure
GPE: Global Perceived Effect
PASS: Patient Acceptable Symptom State
SIRAS: Sports Injury Rehabilitation Adherence Scale
PI: Principal Investigator
SD: Standard Deviation
GLMM: Generalized Linear Mixed Model
