# Supplementary material for: Collaborative model of care between Orthopaedics and allied healthcare professionals in knee osteoarthritis (CONNACT): study protocol for an effectiveness-implementation hybrid randomized control trial
Source: BMC Musculoskelet Disord. 2020 Oct 16;21:684. doi: 10.1186/s12891-020-03695-3 (PMC7568411; doi:10.1186/s12891-020-03695-3)
Supplement: Supplementary file 1 — Appendix 1 – Intervention Description. (DOCX 26 kb) [file 12891_2020_3695_MOESM1_ESM.docx]

|  | **Education** | **Psychological Support** | **Dietetic and Nutrition** | **Exercise Therapy** |
| --- | --- | --- | --- | --- |
| (**Why**)  Describe any rationale, theory or goal of the elements essential to the intervention | Education improves patient satisfaction and coping ability, and reduces unneeded medical services, referrals and expenses. When combined with exercise, education is effective in improving pain, function and quality of life for patients with OA.  Past ‘successful’ patients were invited back as ‘expert patients’ to share their experiences and advices with new patients. The expert patient serves as a testament to 1) cognitive development being the basis of self-management, and 2) successful coping being a combination of acknowledging life with knee osteoarthritis is unpredictable and being realistic, flexible and reasonable. | Acceptance and Commitment Therapy (ACT) can be brief and flexible, yet effective. It has improved functions, quality of life and symptom reduction due to greater acceptance and meaningful engagement in life reliably.  Self-activation helps with long-term self-management, which is a patient’s ability to live as normally with his chronic condition by using precise strategies to cope with symptoms, treatment and effects (i.e. mental, physical, lifestyle and behavioral). It also contributes to a low-cost model of care. | The National Institute for Health and Care Excellence (NICE) recommends interventions to use proven behavior change techniques and align the intervention with the patient’s needs for planning, setting goals and monitoring. To manage obesity, the NICE guideline states: 1) caloric expenditure must exceed caloric intake; 2) a 600kcal deficit diet is effective for weight loss; and 3) a combination of active support for diet and behaviour therapy (problem solving, relapse prevention, stimulus control, dealing with problem situations, assertion, behaviour chain analysis) is effective for weight loss | The American College of Sports Medicine Guidelines recommends at least 150 mins to 300 mins a week of moderate intensity aerobic activity in addition to muscle strengthening activities.    NEuroMuscular EXercise (NEMEX) focuses on lower limb alignment during exercises. Studies have reported feasibility in older end-stage OA patients; reduced pain and training progression; immediate improvement in self-reported outcomes, performance-based measures, and muscle power; and national implementation. |
| **What**  Materials: Describe any physical or informational materials used in the intervention, including those provided to patients or used in intervention delivery or in training of intervention providers. | Printed “Tips for Change” and “Building Resilience” will be given to patients during the first and second sessions respectively. The handouts will be printed in English and Mandarin. | Printed “Fear Avoidance and Hypervigilance” handout will be given during the first session.  During the second session,  printed “Relationships and Communication” handout and a worksheet will be given to patients. A half-full glass and a coin/dollar note were used as materials to teach patients defusion (ACT).  The handouts were printed in English and Mandarin. | Printed “Healthy Eating”, “Label Reading and Calorie Burning” and “Mindful Eating and Self-Monitoring” handouts will be given to patients during each session respectively. The handouts will be printed in English and Mandarin.  To illustrate healthy eating to patients, a Healthy Plate prop will be used during the first session. | A printed exercise handout will be given to patients at the first session.  The exercises, categorized into strengthening, stretching and functional exercises, have simple instructions, illustrations, and the prescribed repetitions and sets to be performed. The handout will be printed in English and Mandarin. |
| **What**  Procedures: Describe each of the procedures, activities, and/or processes used in the intervention, including any enabling or support activities. | **Session 1: Motivate to Activate**  Patients identify pain triggers; relationship between activity, weight and pain; share experiences and struggles with weight/pain management; and set and define behavioral goals. An expert patient will be invited to share his/her experience, struggles, successes and advices with current patients.  **Session 2: Maintaining Good Health for the Long-term**  Patients discuss barriers to and discuss plans for maintaining behavior change; discuss personal times of and develop plans for building resilience; and learn relaxation exercise for visualizing inner strength and reducing physical tension. Re-enforcement of valued living and committed action to goal achievement and managing potential pain flare-ups.  **Support Group Session**  The physician reiterated the concepts taught during the education classes to drive the importance of long-term maintenance and self-management for sustained improvement. The physician also shows the group’s present performance, provide advice for improvement, encourage patients to share and discuss their experience and concerns, and answer questions. | **Session 1: Causes of Pain and Management of Weight/Pain**  Patients learn about fear evasion and hypervigilance; 3Ps (Plan, Prioritize, Pace); and SMART (Specific, Meaningful, Achievable, Realistic, Time-based) short-term goals. They are encouraged to make committed action to the goals they say are important to them They also practice deep-breathing mindfulness, the clipboard metaphor, and plot their knee OA experience on a graph.  **Session 2: Promoting Good Health, Adapting for Leisure and at Work**  Patients will understand and practice cognitive defusion- moving away from what keeps them stuck in effectively managing pain.  Patients will learn how to clarify their values-values clarification of what is important to them and move towards this valued direction than a fixation on getting rid of pain.  They will also recall instances of isolation, quarrels and positive interactions and how they correlate with feelings of increased and decreased pain.  **Session 3: Managing Flare-ups**  Patients will review the tools, learn the steps, and discuss the efficacy and possibilities of strategies for managing flare-ups. They will set new goals or modify them to align with their needs. | **Session 1: General Healthy Eating & Eating Out**  Patients explore their life values and motivations for weight loss. They will learn the benefits of and top 10 tips for healthy weight loss, concept of energy balance, healthy plate composition and portions, healthier options when eating out, and SMART goal-setting.  **Session 2: Label Reading, Snacks & Number of Steps**  Patients will learn label reading, harmful effects of sedentary lifestyles, non-exercise thermogenesis, achieving 500kcal deficit via exercise and diet, and revise their SMART goals.  **Session 3: Mindful Eating, Portion Control and Self-Monitoring**  Patients will learn about mindful eating, the Hunger Scale, reason for eating and portion control, maintaining motivation, and revise their SMART goals. | **Sessions 1-8: Physiotherapy**  Each session begins with flexibility and progresses to strengthening and ends with moderate-intensity aerobic exercises.  The physiotherapist will demonstrate and explain the exercise to the group. Patients will carry out the instructed number of repetitions and sets, while the physiotherapist and assistant supervise closely to correct technique to ensure good alignment based on the NEMEX principles. Patients will be educated on appropriate intensity of exercise based on the Bord scale.  At the end of the class, the physiotherapist recaps the exercises taught and will instructs patients to carry out the exercises at home for sustainable, long-term self-management. |
| **Where**  Describe the location where the intervention occurred, including any necessary infrastructure | The intervention sessions were held at St Luke’s ElderCare, which is a community elder care center. It is conveniently accessible by public transport. The center has a dedicated sizeable space for providing rehabilitation and physiotherapy services to the elderly. The environment is clean, air-conditioned, comfortable and conducive for the intervention. A room is provided for delivering the intervention’s non-physiotherapy classes with minimal intrusion and disruption. The facility is well-equipped with gym machines, light weights and paraphernalia for carrying out the physiotherapy exercises. | | | |
| **When and How Much**  Describe the number of times the intervention was delivered and over what period of time including the number of sessions, their schedule, and their duration, intensity, or dose | **Education**: Two compulsory sessions on week 1 and 12. Each session lasts 1.5 hours.  **Psychological Support**: Three sessions, if prescribed. The sessions were held on week 3, 5 and 7. Each session lasts 1.5 hours.  **Nutrition and Dietetic**: Three sessions, if prescribed. The sessions were held on week 2, 4 and 6. Each session lasts 1.5 hours.  **Exercise Therapy**: Eight compulsory sessions (week 1-7 and 12). Each session will be held after the Education, Psychology and Nutrition and Dietetic sessions. A session lasts 1 hour. Patients will perform the exercises together at a common and manageable intensity and repetition. The physiotherapist will alter the intensity of the exercise(s) based on the individual’s and class’s general performance.  **Support Group**: A session will be held 12 weeks later. The session lasts 1.5 hours. | | | |
| **Who** | Group sessions will be delivered by an Orthopedic Surgeon and a qualified psychologist or medical social work with minimally a Bachelor’s Degree and at least 2 years of experience in delivering behavioral change therapy to chronic pain patients. | Group sessions will be delivered or supervised by a qualified psychologist or medical social worker with minimally a Bachelor’s Degree and at least 2 years of experience in delivering behavioral change therapy to chronic pain patients. | Group sessions will be delivered by a qualified dietitian with minimally a Bachelor’s Degree and at least 2 years of experience in weight management intervention. | Sessions were delivered by a qualified physiotherapist with minimally a Bachelor’s Degree and an assistant with at least 1-year of experience in physiotherapy. |
| **How**  Modes of delivery of the intervention and whether it was provided individually or in a group  Supervision of exercises  Reporting of adherence to exercise  Details of motivation strategies  Decision rules for progressing the exercise program  Content of any home program component | Patients were separated into English and Mandarin language group classes based on their language reference/ability. The classes were held on separate days. Each class has a maximum size of eight patients which optimizes resource utilization without overstraining it. The education, psychology, and nutrition and dietetic classes were administered face-to-face to patients in English or Mandarin respectively based on the language group class they belong to.    The physiotherapy classes were conducted jointly by the lead physiotherapist for both language groups. The physiotherapy sessions were closely monitored by the lead physiotherapist and teaching assistant. They will move about the class and scrutinize the patients’ form, correct mistakes and provide additional assistance, advice or coaching if necessary. Throughout each session, the lead physiotherapist emphasized on the importance of adopting the correct form and maintaining the right alignment based on the NEMEX guidelines when exercising to avoid injuries and reap maximum health benefits for improvement.  During the 3-month, 6-month and 12-month follow-ups, a study team member will administer the Compliance Assessment to Physiotherapy Exercises at Home questionnaire to the patient. The physiotherapist will complete a Sports Injury Rehabilitation Adherence Scale (SIRAS) for each patient after every session.  Strong emphasis on promoting self-management for long term sustainable change is embedded throughout the program through the education and psychology classes grounded in the Acceptance and Commitment Therapy (ACT) principles and Patient Activation strategies. The use of “expert patients” who have previously completed the program will be invited to share his/her experience, struggles, successes and advices with patients. An intact group principle will be kept to promote cohesiveness and peer-support.  If the individual exhibited little/no difficulty and/or pain when performing the exercise, the physiotherapist will progress the exercise’s difficulty by varying its movements, position, or repetitions after accounting for the patient’s condition and pain level. Conversely, the physiotherapist will modify the exercise for individuals who exhibited difficulties and/or pain by simplifying the method, varying the position/movement, and allow the use of equipment (e.g. towel or chair) for support.  After each group physiotherapy session, patients were instructed by the physiotherapist to practice the new exercises that were taught during the session at home. This will be reviewed during the following session. The intervention has a break (week 8 – 11) where patients will exercise at home instead of attending classes to inculcate long-term treatment adherence prior to returning in the final session on week 12. | | | |
| **Tailoring**  Triaging  Whether exercises are generic (“one size fits all”) or tailored to the individual | **Psychological Support**: Prescribed if patient’s PEG > 4 or PHQ-4 > 5, or PAM is Level 1 or Level 2.  **Nutrition and Dietetic**: Prescribed if patient’s Body Mass Index ≥ 23.5.  **Education**: Compulsory for all patients.  **Exercise Therapy**: Compulsory for all patients.  The exercises were performed as a group but tailored to individuals based on their condition and ability. The physiotherapist may progress the exercise’s difficulty for patients who exhibited minimal difficulty/pain when doing the exercise. Conversely, the physiotherapist will simplify exercises for those who experienced greater difficulty/pain by reducing repetitions, changing positions, or use equipment (e.g. towel or chair) for assistance. Patients were also taught modified home exercises by the physiotherapist so that they can perform them at home safely and without the need for specialized equipment. | | | |
