# Supplementary material for: Collaborative model of care between Orthopaedics and allied healthcare professionals in knee osteoarthritis (CONNACT): study protocol for an effectiveness-implementation hybrid randomized control trial
Source: BMC Musculoskelet Disord. 2020 Oct 16;21:684. doi: 10.1186/s12891-020-03695-3 (PMC7568411; doi:10.1186/s12891-020-03695-3)
Supplement: Supplementary file 2 — Appendi× 2 – Patient Reported Exercise Compliance Questionnaire. (PDF 265 kb) [file 12891_2020_3695_MOESM2_ESM.pdf]

Compliance Assessment to Physiotherapy Exercises at home
(Only Applicable to Intervention arm patients)

Following questionnaire will be administered to participants at the 3 month, 6 month and 12 month Follow-up visits to monitor their compliance level at home.

Date of Assessment: ______________

Please circle the most applicable response.

Since the past 3 months

1) On average, how many days did you do the exercises at home
   a. Did not manage to do exercises at all
   b. 1-2 times/week
   c. 3 times/week
   d. 4 times/week
   e. ≥ 5 times/week

2) On average, how many sessions did you perform the exercises each day
   a. None
   b. 1 session/day
   c. 2 sessions/day
   d. 3 sessions/day

3) On average, how many prescribed exercises did you do each session
   a. None
   b. Less than half of the prescribed exercises
   c. Half of the prescribed exercises
   d. More than half of the prescribed exercises
   e. All of the prescribed exercises

4) On average, how many repetitions did you complete for each exercises
   a. None
   b. Less than half of prescribed repetitions
   c. Half of prescribed repetitions
   d. More than half of prescribed repetitions
   e. All prescribed number of repetitions

5) What is the reason(s) for not complying with exercises prescribed at home
   (May circle more than 1 reason)
   a. Unsure of the exercises to do
   b. Time constraint
   c. Pain
   d. Forgetful
   e. Do not believe that therapy will work
   f. Others: ____
