# Supplementary material for: Collaborative model of care between Orthopaedics and allied healthcare professionals in knee osteoarthritis (CONNACT): study protocol for an effectiveness-implementation hybrid randomized control trial
Source: BMC Musculoskelet Disord. 2020 Oct 16;21:684. doi: 10.1186/s12891-020-03695-3 (PMC7568411; doi:10.1186/s12891-020-03695-3)
Supplement: Supplementary file 3 — Appendi× 3 – Cost Questionnaire. (DOCX 43 kb) [file 12891_2020_3695_MOESM3_ESM.docx]

**Modified Osteoarthritis cost and consequences questionnaire**

*** To be administered at baseline, 3 months, 6 months, 9 months and 12 months**

**Employment status**

- Employed (occupation) __________________________
- Unemployed
- Homemaker
- Retired

**Impact on Work**

If you are no longer working, is this because of your knee condition? (Mark ONE)

- Yes
- No

**PATIENT COST**

**If employed,**

If you are in paid work, is this work: (Mark ONE)

- Full time
- Part time

Approximately how many hours do you work per week? (Mark ONE)

- 1 – 10 hours
- 11 – 25 hours
- 26 – 40 hours
- More than 40 hours

Yearly Income bracket (based on IRAS tax bracket)

- Below 20k
- 20k – 30k
- 30k – 40k
- 40k – 80k
- 80k – 120k
- 120k – 160k
- 160k – 200k
- 200k – 240k
- 240k – 280k
- 280k – 320k
- Above 320k

**Absenteeism**

During the past 3 months, how many days did you miss from work because of problems associated your knee condition? Include hours you missed on sick days, medical appointments, times you went in late, left early, etc., because of your knee condition.

_____days *can include half day as well

During the past 3 months, how many days did you miss from work because of any other reason, such as vacation, holidays etc?

_____days *can include half day as well

During the past 3 months, how many days did you actually work?

_____days *can include half day as well

**Presenteeism**

How much did your knee condition affect your productivity on those days you actually worked?

Think about days you were limited in the amount or kind of work you could do, days you accomplished less than you would like, or days you could not do your work as carefully as usual. If your knee condition affected your work only a little, choose a low number. Choose a high number if your knee condition affected your work a great deal.

Consider only how much your knee condition affected your productivity while you were working.

No effect on my work Completely prevented me from working

0 1 2 3 4 5 6 7 8 9 10

(CIRCLE A NUMBER)

**Both employed/unemployed,**

**Non-working activities**

How much did your knee condition affect your ability to do your regular daily activities, other than work at a job?

By regular activities, we mean the usual activities you do, such as work around the house, shopping, childcare, exercising, studying, etc. Think about times you were limited in the amount or kind of activities you could do and times you accomplished less than you would like. If your knee condition affected your activities only a little, choose a low number. Choose a high number if your knee condition affected your activities a great deal.

Consider only how much your knee condition affected your ability to do your regular daily activities, other than work at a job.

No effect on my daily activities Completely prevented me from doing my daily activities

0 1 2 3 4 5 6 7 8 9 10

(CIRCLE A NUMBER)

**PROCEDURES**

What procedures have you had because of your knee condition, in the past three months? (Mark ANY that apply)

- A right knee joint injection?
- A left knee joint injection?
- A right knee joint arthroscopy?
- A left knee joint arthroscopy?
- Other procedures? If so, please specify: _ _ _ _ _ _ _ _ _ _ _ _ _ _ _ _ _ _ _ _ _ _ _ _ _ _
- None of the above

**MEDICATIONS**

In the lists below (A, B & C), please mark which medications you have taken because of your **knee condition** in the past week. For these medications, please – if possible – specify if they were prescribed by a doctor, what dose they are and how much they cost.

A. Please indicate what medications (including over-the-counter medications and herbal supplements) were taken in the past week for your knee condition. (Mark ANY that apply)

| Medication | | How many times  over the past week? |
| --- | --- | --- |
| I take no prescribed medication for my knee condition | € |  |
| Paracetemol e.g. Panadol | € |  |
| Codeine, Dihydricideube, Dextropropoxyphene | € |  |
| Paracetemol & Codeine mix, e.g. Panadeine, codalgin) | € |  |
| NSAID anti-inflammatories, e.g.  Ibuprofen (Neurofen), Brufen, Diclofenac, Voltaren, Cataflam, Naproxen, Naprosyn, Infomethacin | € |  |
| COX-2 inhibitors, e.g. Celecoxib, Celebrex, Etoricoxib, Arcoxia, Lumirocoxib, Prexige, Parecoxib, Dynastate | € |  |
| Tramadol | € |  |
| Oxynom or Oxycodone | € |  |
| Supplements, e.g. Glucosamine, chondroitin | € |  |
| Any other painkillers? If so, please specify:  _________________  _________________ | € |  |

B. Please indicate what medications (including over-the-counter medications and herbal supplements) were taken in the past week for **gastric (stomach) protection**. (Mark any that apply).

| Medication | | How many times  over the past week? |
| --- | --- | --- |
| I take no prescribed medication for my knee condition | € |  |
| Omeprazole e.g. Losec, Omezol | € |  |
| Ranitidine (Zantac) or Famotidine | € |  |
| Any other gastric protection medications? If so, please specify:  _________________  _________________ | € |  |

**ACUTE HEALTH SERVICE USAGE**

Have you visited an accident & emergency department (A&E, or ED) of a hospital because of your treatment for your knee condition in the past three months? (Mark ONE)

- Yes. At Tan Tock Seng Hospital.
- Yes. Other public hospital: Please specify how many times? __
- Yes. Private hospital: Please specify how many times? __
- No

Have you been an in-patient (admitted to hospital overnight) in a hospital because of your knee condition in the past three months? (Mark ONE)

- Yes. At Tan Tock Seng Hospital.
- Yes. Other public hospital.

Please specify how many times? __And, estimate total number of days stayed: __

- Yes. Private hospital: Please specify how many times? __And, estimate total number of days stayed: __
- No

**SPECIALIST / MEDICAL SERVICE USAGE**

How many visits have you made to an **Orthopaedic Surgeon** because of your knee condition in the past three months?

- Tan Tock Seng Hospital (Check EAS system): __
- Other public institution: __
- Private institution: __
- No visits

How many visits have you made to a **Rheumatologist** because of your knee condition in the past three months?

- Tan Tock Seng Hospital (Check EAS system): __
- Other public institution: __
- Private institution: __
- No visits

How many visits have you made to a **Pain Specialist** because of your knee condition in the past three months?

- Tan Tock Seng Hospital (Check EAS system): __
- Other public institution: __
- Private institution: __
- No visits

How many visits have you made to a **General Practitioner (GP)** appointment because of your knee condition in the past three months?

- Yes. Polyclinic: Please specify how many times? __
- Yes. Private GP clinic: Please specify how many times? __
- No visits

How many visits have you made to a **Physiotherapist** appointment because of your knee condition in the past three months?

- Tan Tock Seng Hospital (Check EAS system): __
- Other public institution: Please specify how many times? __
- Private institution: Please specify how many times? __
- No visits

How many visits have you made to a **Dietician** appointment because of your knee condition in the past three months?

- Tan Tock Seng Hospital (Check EAS system): __
- Other public institution: Please specify how many times? __
- Private institution: Please specify how many times? __
- No visits

How many visits have you made to a **Psychologist** appointment because of your knee condition in the past three months?

- Tan Tock Seng Hospital (Check EAS system): __
- Other public institution: Please specify how many times? __
- Private institution: Please specify how many times? __
- No visits

How many visits have you made to an **Occupational Therapist** appointment because of your knee condition in the past three months?

- Tan Tock Seng Hospital (Check EAS system): __
- Other public institution: Please specify how many times? __
- Private institution: Please specify how many times? __
- No visits

How many visits have you made to a **Traditional Chinese Medicine (TCM) Practitioner** because of your knee condition in the past three months?

- Tan Tock Seng Hospital (Check EAS system): __
- Other public institution: Please specify how many times? __
- Private institution: Please specify how many times? __
- No visits

How many times did you receive **X-rays** because of your knee condition in the past three months? (Please place a zero if you had none):

- Tan Tock Seng Hospital (Check EAS system): __
- Other public institution: Please specify how many times? __
- Private institution: Please specify how many times? __
- None
- I do not use any medical services for my knee condition

**COMMUNITY SERVICES**

Please indicate if you have used any of the following services because of your knee condition in the past three months and if so, how many times. (Mark ANY that apply)

- Home help No. of visits? __ Cost to you per visit (if any): $__
- House cleaner No. of visits? __ Cost to you per visit (if any): $__
- Meals on wheels No. of visits? __ Cost to you per visit (if any): $__
- Social worker No. of visits? __ Cost to you per visit (if any): $__
- Day care / Rehabilitation No. of visits? __ Cost to you per visit (if any): $__
- Voluntary organisation helper No. of visits? __ Cost to you per visit (if any): $__
- Other – Please specify: _________ No. of visits? __ Cost to you per visit (if any): $__
- I do not use any health related community services

**TRAVEL COSTS**

Please describe the instances in the last 3 months that you have traveled to attend medical appointments or services (examples described earlier) related to your knee condition (including today).

| Date | Type of appointment / encounter | Mode of transportation | Accompanying Caregiver  ***please indicate if more than 1** | Time spent (including travel) | Other costs? |
| --- | --- | --- | --- | --- | --- |
|  | - Ortho - PT - Dietician - Psychologist - OT - ED visit - Inpatient admission - GP - Others: ________ | - Car - Taxi - Bus - MRT - Ambulance - Not Applicable (e.g. by walking) | - FDW - Non-working family member - Working family member - Not Applicable |  | - Parking - Others:   ______ |
|  | - Ortho - PT - Dietician - Psychologist - OT - ED visit - Inpatient admission - GP - Others: ________ | - Car - Taxi - Bus - MRT - Ambulance - Not Applicable (e.g. by walking) | - FDW - Non-working family member - Working family member - Not Applicable |  | - Parking - Others:   ______ |
|  | - Ortho - PT - Dietician - Psychologist - OT - ED visit - Inpatient admission - GP - Others: ________ | - Car - Taxi - Bus - MRT - Ambulance - Not Applicable (e.g. by walking) | - FDW - Non-working family member - Working family member - Not Applicable |  | - Parking - Others:   ______ |

|  | - Ortho - PT - Dietician - Psychologist - OT - ED visit - Inpatient admission - GP - Others: ________ | - Car - Taxi - Bus - MRT - Ambulance - Not Applicable (e.g. by walking) | - FDW - Non-working family member - Working family member - Not Applicable |  | - Parking - Others:   ______ |
| --- | --- | --- | --- | --- | --- |
|  | - Ortho - PT - Dietician - Psychologist - OT - ED visit - Inpatient admission - GP - Others: ________ | - Car - Taxi - Bus - MRT - Ambulance - Not Applicable (e.g. by walking) | - FDW - Non-working family member - Working family member - Not Applicable |  | - Parking - Others:   ______ |
|  | - Ortho - PT - Dietician - Psychologist - OT - ED visit - Inpatient admission - GP - Others: ________ | - Car - Taxi - Bus - MRT - Ambulance - Not Applicable (e.g. by walking) | - FDW - Non-working family member - Working family member - Not Applicable |  | - Parking - Others:   ______ |

For Intervention Arm at 3 months Follow-up:

| Location | Dates | Type of appointment / encounter | Mode of Transportation | Accompanying Caregiver  ***please indicate if more than 1** | Time spent (including travel) | Other costs? |
| --- | --- | --- | --- | --- | --- | --- |
| Community-based Rehabilitation  Facility  No. of times  PT: _______  NND: _____  PSY: ______ | 1 __________  2 __________  3 __________  4 __________  5 __________  6 __________  7 __________  8 __________ | - PT - Dietician - Psychologist | - Car - Taxi - Bus - MRT - Ambulance - Not Applicable (e.g. by walking) | - FDW - Non-working family member - Working family member - Not Applicable |  | - Parking - Others:   ______ |

Address (postal code): _________________

Distance to hospital __________________ (km) *refer to Googlemaps for calculation

**AIDS AND ADAPTATIONS**

Have you purchased or been prescribed aids (bath/toilet aids, walking sticks etc.) to help with your knee condition in the past 3 months? (Mark ANY that apply)

- Walking stick
- Walker frame
- Rollator frame
- Reacher (helping hand)
- Toilet grab bar / toilet frame
- Shower chair
- Sock aide
- Knee guard
- Others. If so, please specify:
- _ _ _ _ _ _ _ _ _ _ _ _ _ _ _ _ _ _ _ _ _ _ _ _ _ _ _ _ _ _ _ _ _ _ _ _
- No, none purchase or prescribed

Have you made adaptations to your home or lifestyle (other than ones discussed in this questionnaire) because of your knee condition in the past 3 months? (Mark ONE)

- None
- If yes, please indicate what adaptation(s) you have made and the estimated cost:

_ _ _ _ _ _ _ _ _ _ _ _ _ _ _ _ _ _ _ _ _ _ _ _ _ _ _ _ _ _ _ _ _ _ _

If there are other costs or consequences of your knee condition, or if you have any comments you would like to share with us regarding any aspect of the CONNACT study, please provide them in the space below.

_ _ _ _ _ _ _ _ _ _ _ _ _ _ _ _ _ _ _ _ _ _ _ _ _ _ _ _ _ _ _ _ _ _ _ _ _ _ _ _ _

_ _ _ _ _ _ _ _ _ _ _ _ _ _ _ _ _ _ _ _ _ _ _ _ _ _ _ _ _ _ _ _ _ _ _ _ _ _ _ _ _

Data Collected by:

Name, Signature and Date

Name, Signature and Date
