# Supplementary material for: Collaborative model of care between Orthopaedics and allied healthcare professionals in knee osteoarthritis (CONNACT): study protocol for an effectiveness-implementation hybrid randomized control trial
Source: BMC Musculoskelet Disord. 2020 Oct 16;21:684. doi: 10.1186/s12891-020-03695-3 (PMC7568411; doi:10.1186/s12891-020-03695-3)
Supplement: Supplementary file 4 — Appendix 4 – SPIRIT diagram. (DOC 74 kb) [file 12891_2020_3695_MOESM4_ESM.doc]

Figure 2 SPIRIT diagram

|  | **STUDY PERIOD** | | | | | |
| --- | --- | --- | --- | --- | --- | --- |
|  | **Enrolment** | **Allocation** | **Post-allocation** | | | **Close-out** |
| **TIMEPOINT(MONTHS)** |  |  | **3 months** | **6 months** | **9 months** | **12 months** |
| **ENROLMENT:** |  |  |  |  |  |  |
| Eligibility screen | x |  |  |  |  |  |
| Informed Consent | x |  |  |  |  |  |
| Randomization |  | x |  |  |  |  |
| **INTERVENTIONS:** |  |  |  |  |  |  |
| CONNACT Model of Care |  |  |  |  |  |  |
| Usual Care |  |  |  |  |  |  |
| **ASSESSMENT:** |  |  |  |  |  |  |
| Baseline variables | x | x |  |  |  |  |
| Demographics | x | x |  |  |  |  |
| Socioeconomic status | x | x |  |  |  |  |
| Co-morbidities and functional status | x | x |  |  |  |  |
| Knee symptoms and duration | x | x |  |  |  |  |
| Radiographic severity (Kellgren Lawrence Scale) | x | x |  |  |  |  |
| Effectiveness Measures: |  |  |  |  |  |  |
| Knee Osteoarthritis and Outcome Score (KOOS) |  | x | x | x |  | x |
| Functional Performance |  | x | x | x |  | x |
| UCLA Activity Score |  | x | x | x |  | x |
| Body Mass Index |  | x | x | x |  | x |
| Modified Food Frequency Questionnaire (FFQ)* |  | x | x | x |  | x |
| Pain Interference, Enjoyment, General Activity Scale (PEG) |  | x | x | x |  | x |
| Patient Health Questionnaire-4 (PHQ-4) |  | x | x | x |  | x |
| Patient Activation Measure (PAM) |  | x | x | x |  | x |
| EQ-5D |  | x | x | x |  | x |
| Analgesia Consumption |  |  | x | x |  | x |
| Global Perceived Effect (GPE) |  |  | x | x |  | x |
| Patient Acceptable Symptom Score (PASS) |  |  | x | x |  | x |
| Compliance and Adherence Measures (intervention arm patients) |  |  |  |  |  |  |
| Exercise Compliance Questionnaire |  |  | x | x |  | x |
| Sports Injury Rehabilitation Adherence Scale# |  |  | x |  |  |  |
| **ECONOMIC EVALUATION:** |  |  |  |  |  |  |
| Cost Questionnaire |  |  | x | x | x | x |
| **QUALITATIVE:** |  |  |  |  |  |  |
| Patient Interviews |  |  | x |  |  | x |

*only patients with BMI > 23.5

#done after every physiotherapy session
